# Social health markers in the context of cognitive decline and dementia: an international qualitative study

**DOI:** 10.3389/fpsyt.2024.1384636

**Published:** 2024-09-19

**Authors:** Martina S. Kristanti, Myrra Vernooij-Dassen, Yun-Hee Jeon, Eline Verspoor, Suraj Samtani, Giovanni Ottoboni, Rabih Chattat, Henry Brodaty, Marta Lenart-Bugla, Krzysztof Kowalski, Joanna Rymaszewska, Dorota M. Szczesniak, Ansgar Gerhardus, Imke Seifert, Muhamad Zulvatul A’la, Christantie Effendy, Marieke Perry

**Affiliations:** ^1^ Department of Basic and Emergency Nursing, Faculty of Medicine, Public Health and Nursing, Universitas Gadjah Mada, Yogyakarta, Indonesia; ^2^ Department of IQ Healthcare, Radboud University Medical Centre, Nijmegen, Netherlands; ^3^ Sydney Nursing School, The University of Sydney, Darlington, NSW, Australia; ^4^ Department of Geriatrics, Radboud University Medical Centre, Nijmegen, Netherlands; ^5^ Centre for Healthy Brain Ageing, School of Psychiatry, Faculty of Medicine, University of New South Wales, Randwick, NSW, Australia; ^6^ Department of Psychology, University of Bologna, Bologna, Italy; ^7^ Department of Psychiatry, Wroclaw Medical University, Wroclaw, Poland; ^8^ Department of Clinical Neuroscience, Faculty of Medicine, Wroclaw University of Science and Technology, Wroclaw, Poland; ^9^ Department for Health Services Research, Institute for Public Health and Nursing Research, University of Bremen, Bremen, Germany; ^10^ Faculty of Nursing, University of Jember, Jember, Indonesia; ^11^ Department of Medical Surgical Nursing, Faculty of Medicine, Public Health and Nursing, Universitas Gadjah Mada, Yogyakarta, Indonesia

**Keywords:** social health, social health markers, qualitative, multicountry analysis, dementia - alzheimer disease

## Abstract

**Background:**

Social health in the context of dementia has recently gained interest. The development of a social health conceptual framework at the individual and social environmental levels, has revealed a critical need for a further exploration of social health markers that can be used in the development of dementia intervention and to construct social health measures.

**Objective:**

To identify social health markers in the context of dementia.

**Method:**

This international qualitative study included six countries: Australia, Germany, Indonesia, Italy, Poland, and the Netherlands. Using purposive sampling, three to five cases per country were recruited to the study, with each case consisting of a person living with dementia, a primary informal caregiver, an active network member, and a health care professional involved in the care of the person with dementia. In-depth interviews, using an agreed topic guide, and content analysis were conducted to identify known and new social health markers. The codes were then categorized against our conceptual framework of social health.

**Results:**

Sixty-seven participants were interviewed. We identified various social health markers, ranging from those that are commonly used in epidemiological studies such as loneliness to novel markers of social health at the individual and the social environmental level. Examples of novel individual-level markers were efforts to comply with social norms and making own choices in, for example, keeping contact or refusing support. At a social environmental level, examples of novel markers were proximity (physical distance) and the function of the social network of helping the person maintaining dignity.

**Conclusions:**

The current study identified both well-known and novel social health markers in the context of dementia, mapped to the social health framework we developed. Future research should focus on translating these markers into validated measures and on developing social health focused interventions for persons with dementia.

## 1 Introduction

Dementia is acknowledged as a syndrome with multifactorial causes and multiple presentations. In relation to its development, focus on social health has recently gained interest in addition to biological (1–4) and psychological factors (5, 6). In its presentation, dementia profoundly affects the individual’s life, not only cognitively, physically and mentally, but also socially (7). Changes in social functioning often are the first signs of dementia (8, 9). During the trajectory of dementia, persons with dementia struggle with their social identity and can feel like outcasts (10). Dementia changes communication abilities and opportunities (11) and is associated with a reduction in social network size (12–15). Qualitative studies have shown how persons with dementia strive to maintain their social connections (16, 17). This illustrates the importance of social health in the context of dementia.

Social health is an umbrella concept: ‘*It is essentially a relational concept in which wellbeing is defined on the one hand, as the impact that an individual has on others (social environment), and on the other hand as the impact that the social environment (others) has, in turn, on the individual*’ (18). Social health in the context of dementia is seen as a new perspective on maintaining potential and capacity. We used the conceptual framework for social health in the context of dementia which we developed in our previous work to organize social health markers on its individual and social environmental levels (18). The next step is to identify relevant social health markers to enable measurement of social health.

The conceptual framework (18) can be used to study relationships between social health markers and cognitive functioning. Epidemiological studies have already demonstrated associations between specific social health markers (e.g., social support, social participation) and cognitive functioning and dementia risk (1, 2, 4, 19–21). A higher level of social connectivity was associated with lower cognitive decline (3, 20) and lower risk of dementia (21–23). Conversely, loneliness (1) and the lack of people to talk with on a daily basis (5, 6) were associated with cognitive decline and risk of dementia.

Both the framework (18) and epidemiological research however lack a clear articulation of relevant social health markers. Especially social health markers on the level of the individual are narrowly defined in the domains of individual’s autonomy and their capacity to fulfill their potential. Markers on the social environmental level mainly focus on the domains of network structure and social support (18). The current qualitative research aims to identify new social health markers in the context of dementia, relevant to people with dementia and their social environment. This will facilitate a better understanding of social health. New social health markers can be used for consideration of potentially modifiable protective and risk factors for development of novel interventions as well as for construction of social health measurement.

## 2 Method

### 2.1 Study context

This study was conducted as part of the ‘Social Health And Reserve in the Dementia patient journey (SHARED)’ project involving diverse disciplines including epidemiology, sociology, psychology, nursing, geriatrics, psychiatry, neuropsychiatry, and family practice. The SHARED project aims to identify the theoretical and epidemiological associations between social health markers, cognitive reserve, and dementia risk in older adults. The social health conceptual framework (18) has been developed as part of the SHARED project.

### 2.2 Design and setting

The study is an international qualitative study using in-depth interviews. It was conducted in: Australia (AU), Germany (GE), Indonesia (ID), Italy (IT), the Netherlands (NL), and Poland (PL). All countries had at least one researcher in their team who was experienced in conducting qualitative research and dementia research. The Consolidated Criteria for Reporting Qualitative studies (COREQ) checklist was used for reporting (24) (**Appendix 2**).

### 2.3 Participants and recruitment

In each of the six countries, we aimed to recruit quartets consisting of person with dementia, and an associated primary informal caregiver, active network member, and health care professional. We employed purposive sampling. In the first step, participants were recruited based on inclusion and exclusion criteria (**Table 1**).

**Table 1:** Information of inclusion and exclusion criteria.

Participants	Inclusion	Exclusion
Person with Dementia	- Community-dwelling older adult aged 60 years and over. - Dementia diagnosis (type of dementia: Alzheimer’s disease, Lewy body or Vascular dementia). - Is able to consent with participation an interview themselves. - Has language skills to participate in interviews. - Agrees that their primary informal caregiver, primary healthcare professional and one other key network member will be approached to participate in the study.	- Person with dementia living in an assisted living facility (e.g.: nursing home) - Person with dementia whose treating doctor considers such an interview too burdensome (if possible). - Person with dementia with a life expectancy of less than 3 months. - Person with dementia with comorbid severe physical or mental illness that would prevent them from completing the interview.
Direct social network: Primary informal caregiver	- Person who takes responsibility to take care for persons with dementia (e.g., partner, sibling, child, neighbor)	- Informal caregiver, healthcare professional, or other key network member will be excluded when they have a severe physical or mental illness or dementia that would prevent them from completing the interview.
Direct social network: Active network member	- Person from the persons with dementia’s network (e.g., family member, neighbor or friend)	
Direct social network: Health care professional	- Healthcare professional who is involved in the care for the persons with dementia (e.g., case manager, general practitioner).	

In the second step, we strived for a country level maximum variation of sampling taking into account age, gender, living condition (alone/with family) of the person with dementia, and types of health care professionals.

### 2.4 Sample size

In this international study, we aimed to identify social health markers for persons with dementia from the perspective of persons with dementia and their key social network members, irrespective of culture or socio-economic status. We therefore did not aim to achieve data saturation in each participating country, but to achieve data saturation based on the combined data of all participating countries, which we expected after three cases per country (25).

### 2.5 Data collection

We employed in-depth interviews guided by a participant recruitment and data collection protocol developed by the NL team. Potential participants were approached either at clinics or general practices (NL, PL and ID), or utilizing patient support networks namely Deutsche Alzheimer Gesellschaft e.V. (GE), StepUp for Dementia Research (AU) (26) and Alzheimer Café (IT). In each country, the interviews were led by experienced interviewers in their local languages (EV in NL, ML in PL, IS in GE, GO in IT, SS in AU and MSK in ID) (27).

We collected data in 2020-2021 while COVID-19 public health orders still varied amongst countries. NL and PL collected data using in-person meetings while others relied on videocalls for data collection. Participants were interviewed once only and all interviews were audio recorded. Interviewers did not know the participants prior to data collection.

The interview topic guide was developed based on a multi-country qualitative study (28), and experiences from all authors, and subsequently tested in NL prior to data collection and further refined. It included items on dementia diagnosis, social network and daily activities, influence of social network on functioning, changes in social network and interaction with social network after dementia diagnosis and influence of functioning on social network (**Appendix 1**).

We also collected demographic characteristics from all participants. In order to provide more information on the context of persons with dementia social engagement, social network characteristics of persons with dementia were explored and described as high, medium and poor levels per case, based on the individual’s frequency of contacts, number of people seen, appreciation, contact with the community and feeling of being socially isolated.

### 2.6 Data analysis

We used qualitative content analysis (29). Audio recordings were transcribed verbatim using each country’s local language. In order to enhance the feasibility to communicate to all participating countries, the transcripts were coded in English (27). To enhance objectivity, we had at least two researchers in each country who conducted coding and discussed amongst each other.

Data analysis was conducted iteratively using a codebook developed by the NL team in collaboration with all other participating countries. The codebook consisted of codes including definitions describing what is and what it is not in the scope of the code. All participating countries conducted open coding inductively. They were able to use and add codes to the codebook during the coding process, when necessary. Next, multiple meetings amongst all participating countries were conducted to iteratively revise the codebook. Codes were considered markers (**Table 2**). After consensus on coding was reached, we used the conceptual framework of social health (18) to cluster the codes into categories (equal with *domains* in the framework: **Table 2**) and left the possibility to formulate new categories when necessary.

**Table 2:** Qualitative data analysis terminology used in the current study.

Qualitative terminology	Equivalent concept used in the social health framework	Examples
Theme	Level	Individual vs Environmental
Category	Domain	The capacity to fulfil one’s potential and obligations
Sub category	Sub-domain	Comply with social norms
Code	Marker	Person with dementia motivated to comply with social norms

The discussion ended when the consensus for coding and categorization was reached. During the coding process, some participating countries used Atlas.ti (NL, ID) to organize data while others used NVivo (AU), MAXQDA (GE) and Excel (IT, PL). Further we utilized Excel spreadsheets and an online application (Miro https://miro.com) to facilitate team discussion and categorization.

### 2.7 Ethical consideration

All countries used equivalent protocols and adapted to be submitted to ethical committee in each country (30). Ethical permission received from Australia (Ref: HC210100), Germany (ref. No: 2021-14/06-3), Indonesia (Ref: KE/KF/0230/ES/2021), Italy (Ref: Prot.n.170353), Poland (Ref: KB-162/2021) and the Netherlands (Ref: 2020-6153).

## 3 Results

### 3.1 Participant characteristics

Of a total of 20 cases (*n*=67 participants), eight cases were complete members of four, 11 cases included three members each (8 cases without healthcare professionals and 3 cases without other network members) and 1 case was represented by two, a person with dementia and the informal caregiver. **Table 3** provides detailed characteristics of individual cases and participants.

**Table 3:** Characteristics of persons with dementia and the direct social networks.

Case	ID	Sex	Age	Type of dementia	Years since diagnosis	Civil status	Living situation	Social network	Direct social network members	Sex	Age	Kinship with Person with Dementia
1	AU1	F	70s	Alzheimer’s	2	Married	With spouse	Medium	Primary informal caregiver	M	70s	Partner
									Active network member	F	70s	Friend
2	AU2	M	70s	Vascular dementia	1	In a relationship	With partner	Medium	Primary informal caregiver	F	60s	Partner
									Active network member	F	30s	Partner’s daughter
									Health care professional	F	50s	Dementia care coordinator
3	AU3	M	70s	Alzheimer’s	3	Married	With spouse	High	Primary informal caregiver	F	70s	Partner
									Active network member	M	40s	Son
4	GE1	F	80s	Non-specified dementia	unknown	Married	With spouse	Medium	Primary informal caregiver	M	90s	Partner
									Active network member	F	60s	Daughter
5	GE2	M	50s	Alzheimer’s	3	Married	Alone	High	Primary informal caregiver	F	60s	Partner
									Active network member	M	20s	Son
6	GE3	F	60s	Lewy Body	4	Single	With cousin	High	Primary informal caregiver	F	30s	Daughter
									Active network member	F	30s	Daughter
7	ID1	M	70s	Alzheimer’s	5	Married	With spouse	Medium	Primary informal caregiver	F	50s	Wife
									Active network member	M	20s	Son
									Primary health care professional	F	50s	Neurologist
8	ID2	M	60s	Alzheimer’s	3	Married	With spouse	High	Primary informal caregiver	F	60s	Wife
									Active network member	F	30s	Daughter in law
									Primary health care professional	F	50s	Neurologist
9	ID3	M	60s	Vascular dementia	4	Married	With spouse	High	Primary informal caregiver	F	60s	Wife
									Active network member	F	40s	Sister
									Primary health care professional	F	50s	Neurologist
10	ID4	M	70s	Alzheimer’s	5	Married	With spouse	High	Primary informal caregiver	F	70s	Wife
									Active network member	M	40s	Son in law
11	ID5	M	60s	Alzheimer’s	3	Married	With spouse	High	Primary informal caregiver	F	60s	Wife
									Active network member	F	30s	Daughter
12	IT1	F	70s	Alzheimer’s	2	Married	With spouse	High	Primary informal caregiver	M	70s	Partner
									Active network member	F	70s	Friend
13	IT2	M	70s	Progressive aphasia	7	Married	With spouse	High	Primary informal caregiver	F	70s	Partner
									Active network member	M	70s	Friend
									Primary health care professional	M	70s	GP
14	IT3	M	80s	Alzheimer’s	7	Married	With spouse	High	Primary informal caregiver	F	70s	Partner
15	PL1	F	80s	Non-specified dementia	Many years	Single	Alone	Poor	Primary informal caregiver	F	80s	Cousin
									Primary health care professional	F	40s	Psychiatrist
16	PL2	F	80s	Non-specified dementia	2	Widower	With spouse	Poor	Primary informal caregiver	F	60s	Daughter
									Primary health care professional	F	50s	Psychiatrist
17	PL3	F	60s	Non-specified dementia	5	Married	With spouse	Medium	Primary informal caregiver	M	70s	Husband
									Primary health care professional	F	40s	Psychiatrist
18	NL1	M	70s	Alzheimer’s	4	Widower	With son	High	Primary informal caregiver	M	50s	Son
									Active network member	F	50s	Sister-in-law
									Primary health care professional	F	60s	Case Manager
19	NL2	M	80s	Alzheimer’s	3	Married	With spouse	Medium	Primary informal caregiver	F	80s	Wife
									Active network member	M	40s	Healthcare student
									Primary health care professional	F	50s	Case Manager
20	NL3	F	70s	Alzheimer’s	3	Married	With spouse	Medium	Primary informal caregiver	F	60s	Husband
									Active network member	F	60s	Sister
									Primary health care professional	F	50s	Case Manager

AU = Australia; GE = Germany; ID = Indonesia; IT = Italy; PL = Poland; NL = The Netherlands.

Exact age is not provided to protect potential identification of individual participants.

Twenty persons with dementia were interviewed: 12 males and 8 females. The average age was 72.8 years (range: 61 – 88 years). They were diagnosed with Alzheimer’s Disease (n=12), vascular dementia (n=2), Lewy body dementia (n=1), progressive aphasia (n=1), or the non-specific type of dementia (n=4). Two persons with dementia lived alone, all others lived either with their spouse/partner (n=16), adult children (n=1) or other family members (n=1). Time from diagnosis ranged from 1 to 7 years (there were also 2 cases of ‘many years’ and ‘unknown). The social network levels of the participating persons with dementia varied: high (n=11), medium (n=7), and poor (n=2).

Of 20 primary informal caregivers, 14 were female and 15 were spouses. The mean age of the primary informal caregivers was 67 years. Sixteen active network members were involved in the study and eight were adult children of those people with dementia. They had a mean age of 48 years. Eleven health care professionals were involved in the current study. They were managers (*n*=3), psychiatrists (*n*=3), neurologists (*n*=2), a General Practitioner (*n*=1), and a dementia care coordinator (*n*=1).

### 3.2 Identification of social health markers

Iterative collaborative analyses yielded 47 codes/social health markers. All markers were used multiple times in the participating countries (**Table 4**).

**Table 4:** List of markers and its appearance in data of participating countries.

No	Codes/Markers	AU	ID	IT	GE	PL	NL
1	Acceptance of support	X		X	X	X	X
2	Affection	X		X	X	X	X
3	Changing social roles		X		X		
4	Closeness of relationships	X		X			
5	Emotional support	X	X	X	X	X	X
6	Engaging in activities	X	X				
7	Social network appreciating persons with dementia			X	X	X	X
8	Social network avoids contact with persons with dementia	X			X	X	X
9	Social network does/does not criticize/correct persons with dementia	X		X		X	X
10	Social network does/does not involve persons with dementia in conversation				X	X	X
11	Social network does/does not involve persons with dementia in decision-making		X		X	X	X
12	Social network encourages/limits persons with dementia	X	X	X	X	X	X
13	Social network maintaining dignity of persons with dementia	X	X				
14	Social network: Reaction/ dealing with the symptoms/diagnosis		X		X		
15	Instrumental/practical support		X		X		X
16	Limiting independence - negative emotions	X				X	
17	Live life as usual			X	X		
18	Loss of grip	X	X		X	X	X
19	Loss of initiative			X	X	X	X
20	Normalising dementia	X	X	X			
21	Proximity (physical distance) to social contacts	X	X	X			
22	Persons with dementia able/unable to engage in social/daily activities independently	X				X	
23	Person with dementia adapts/does not adapt	X	X	X	X	X	X
24	Persons with dementia appreciating Social network			X	X	X	X
25	Persons with dementia can/cannot maintain contacts/relationships	X				X	
26	Person with dementia does/does not show demanding behavior					X	
27	Person with dementia does/does not want to preserve autonomy of contacts and activities				X	X	X
28	Persons with dementia engaging in decision planning	X	X				
29	Person with dementia enjoys/does not enjoy social interactions	X			X	X	X
30	Person with dementia feels like they are a burden on social network				X	X	X
31	Person with dementia feels that dementia (problems with walking, understanding) makes live contacts difficult	X				X	
32	Person with dementia finds it easy/difficult to trust others	X		X	X	X	X
33	Person with dementia has less energy for contacts and activities		X	X	X	X	X
34	Person with dementia has/does not have ability to engage in conversations	X			X	X	X
35	Person with dementia having empathy for others	X	X				
36	Person with dementia is alone / lacks social contacts	X		X	X	X	X
37	Persons with dementia motivated to comply with social norms	X			X	X	X
38	Persons with dementia receives/does not receive professional support	X		X	X		
39	Persons with dementia withdrawing from/during social activities	X			X	X	X
40	Reciprocity between persons with dementia and social network			X	X	X	X
41	Relationship/marital status	X	X				
42	Shared common ground		X	X	X		
43	Sharing experiences			X	X	X	X
44	Shielding	X		X	X	X	X
45	Social network of person with dementia		X		X		
46	Support of people with lived experience of dementia		X	X			
47	Using humour	X	X		X		X

AU = Australia; GE = Germany; ID = Indonesia; IT = Italy; PL = Poland; NL = The Netherlands.

X = the code exists in the interview transcripts within the sample.

**Figure 1:**
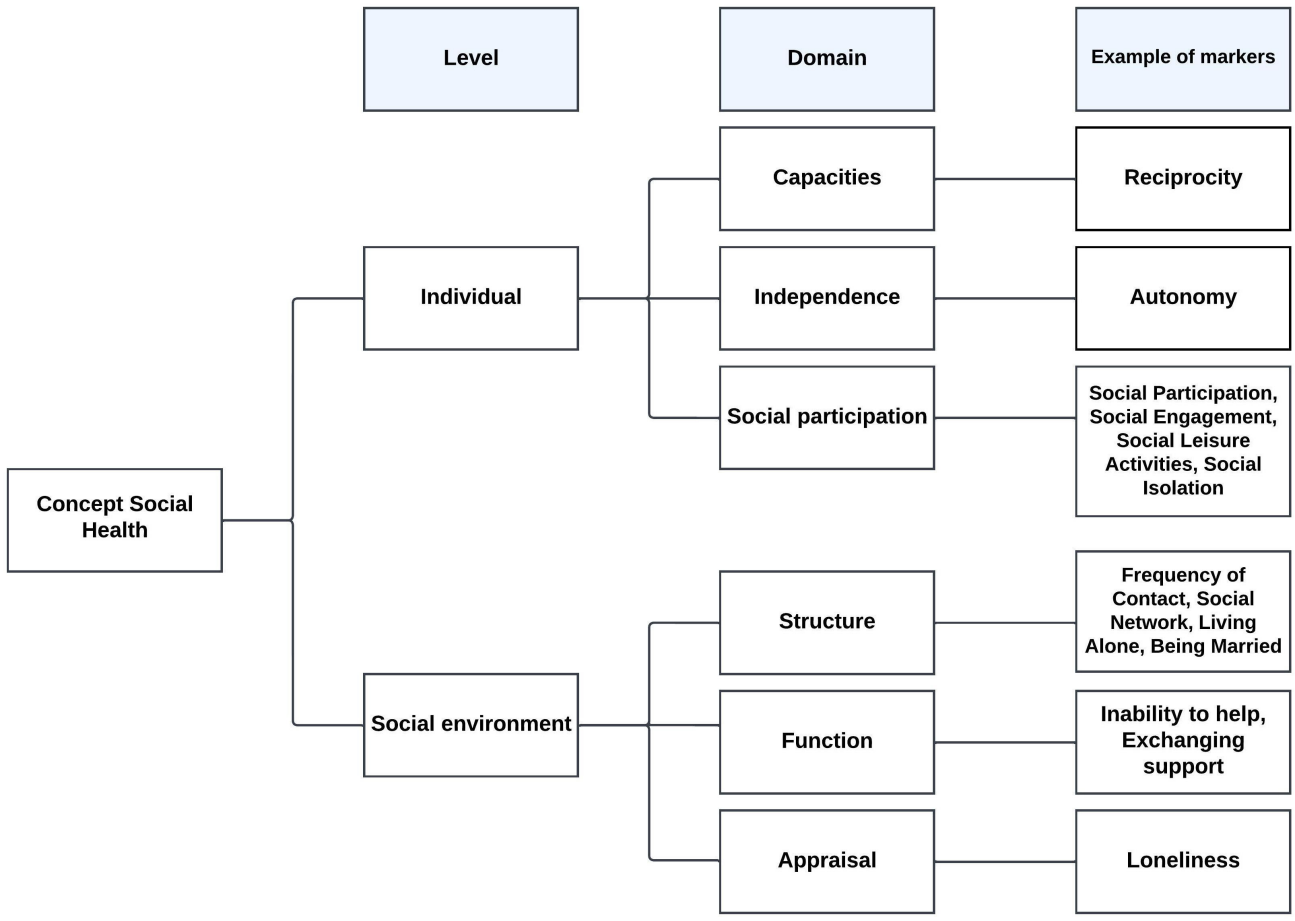
Conceptual framework of social health (18).

The markers were organized into six categories that are equivalent with the domains of the conceptual framework (18): capacities, independence, social participation, structure, function and appraisal (**Figure 1**). These domains are subsequently clustered into two levels: the individual and the social environmental. **Table 5** provides an overview of social health markers, and domains. In this table, we also indicated some social health markers that have not used in previous epidemiological nor intervention studies.

**Table 5:** Social health markers of persons with dementia.

Social health markers	Sub-domain	Domain	Level
*Person with dementia motivated to comply with social norms	Comply with social norms	The capacity to fulfil one’s potential and obligations	Individual
Live life as usual			
Reciprocity between persons with dementia and social network			
*Person with dementia having empathy for others			
Person with dementia finds it easy/difficult to trust others	Dementia symptoms limit social capacity		
Person with dementia does/does not show demanding behavior			
Loss of initiative			
Person with dementia has less energy for contacts and activities			
Loss of grip/self esteem			
*Person with dementia adapts/does not adapt	Adaptation		
Person with dementia feels like they are a burden on social network			
Person with dementia feels that dementia (problems with walking, understanding) makes live contacts difficult			
Person with dementia does/does not want to preserve autonomy of contacts and activities	Choices for some degree of involvement keep contact/ decision planning	The ability to manage life with some degree of independence	
*Person with dementia engaging in decision planning			
*Acceptance of support	Choice to accept/not to accept support		
*Person with dementia receives/does not receive professional support			
*Changing social roles	Adjustment of social roles		
Engaging in activities	Degree of participation	Ability to actively participate in social activities	
Person with dementia withdrawing from/during social activities			
Person with dementia can/cannot maintain contacts/relationships	Reasons not to engage		
Person with dementia has/does not have ability to engage in conversations			
Person with dementia able/unable to engage in social/daily activities independently			
Social network of person with dementia	Structure	Structure of social network	Social environ-mental
Relationship/marital status			
Person with dementia is alone / lacks social contacts			
*Proximity (physical distance) to social contacts			
Instrumental/practical support	Types of support	Function of social network	
Emotional support			
Sharing experiences			
*Using humour			
Providing structure to person with dementia			
Support of friends/peers with living/lived experience	Sources		
*Social network maintaining dignity of persons with dementia	Maintain Dignity		
*Shielding			
Normalising dementia			
Social network does/does not criticize/correct person with dementia			
Social network does/does not involve persons with dementia in conversation	Nonconstructive actions		
Social network avoids contact with persons with dementia			
Limiting independence - negative emotions			
Social network: Reaction/ dealing with the symptoms/diagnosis			
Indifference of the social network makes it difficult to cope with the disease			
Affection	Affection	Appraisal of relationship	
Closeness of relationships			
Shared common ground			
Person with dementia enjoys/does not enjoy social interactions			
Person with dementia appreciating social network	Appreciation		
Social network appreciating persons with dementia			

*New social health markers identified in the current study.

Below, we describe the storyline on markers of social health for persons with dementia in the level domains and sub-domains.

### 3.3 Individual level

#### 3.3.1 Capacity to fulfill one’s potential and obligations

Persons with dementia described how they used their talents and skills to comply with social norms, in order to maintain social engagement. They did so by making efforts to *live their life as usual*, for example, by visiting friends, taking a walk, doing grocery shopping, preparing their own meals or visiting the physician independently when it was possible. As part of living life as usual, persons with dementia found it important to show empathy to others.

“*I’d like to pay sometimes for the one [refer to persons with dementia’s friend] who didn’t have much money with him*.” (Person with dementia, The Netherlands)

Persons with dementia identified *reciprocity* as an important social norm: if you get something, you want to give something back. Despite their limitations, persons with dementia expressed their desire to give something to others.

“*She (the neighbor) says I’m good and helpful to her. I kept her spirits up while she was in the hospital*.” (Person with dementia, Poland)

Persons with dementia and member of their social network mentioned several dementia symptoms and characteristics that limited the capacity to comply with social norms. As part of the condition, persons with dementia had issues on trusting others. It was reported that persons with dementia tended to be more suspicious toward others, even towards their close ones, for example:

“*I myself start to think that people may think that I have a problem on my mind and that they think: ‘You don’t have to talk to that man because he doesn’t know anything anymore’.*” (Person with dementia, The Netherlands)

“*She (person with dementia) became more sensitive and feels mistreated easily*.” (Health care professional, Poland)

Persons with dementia also lost some skills and physical functions, therefore they worried about being a burden to their social environment and were frustrated not to able to maintain the capacity they used to have. Dementia had affected them physically so that they lost energy for contacts and activities. Persons with dementia claimed that their loss of energy might have contributed to the fact that they became less active and lost initiative. With those changes, persons with dementia reported to lose their confidence and self-esteem.

Persons with dementia also reported feeling frustration.

“*Sometimes I get lost in chaos because I can’t get certain things structured, sorted and then it looks as if a bomb has fallen in*.” (Person with dementia, Germany)

Despite these limitations, persons with dementia emphasized their continuous efforts and perseverance to adapt to their current capabilities and health status, also in order to continue to comply with social norms and remain independent.

“*I fight every day to be independent, to be self-reliant*.” (Person with dementia, Germany)

“*I write everything down on sticky notes and put it on the front of fridge*.” (Person with dementia, Poland)

#### 3.3.2 The ability to manage life with some degree of independence

Persons with dementia described several aspects of autonomy, which is about the state of being self-governing and to solve problems in life. We found that persons with dementia used their autonomy to make decisions on whether they want or do not want to preserve contacts or activities or to engage in decision planning. Also, we found that persons with dementia carefully weighed their capability to decide what daily activities to perform and how to perform these, to remain as independent as possible.

“*What is still possible is to go out for a short time up and down the street. Not too far from home. To the bakery is fine. That’s not too far either. To the doctor … that’s also within walking distance*.” (Person with dementia, Germany)

We also identified that persons with dementia used their autonomy to make decisions on professional and social support that was offered. Persons with dementia realized that that they can either accept or decline.

“*I appreciate he (carer) takes me to the doctor, since I’m not sure I can write everything down and understand by myself*.” (Person with dementia, Poland)

Persons with dementia made certain choices in life that changed their social roles. Like in one case, a person with dementia who was a leader in a social organization, decided to resign his position as a leader and continue to stay in the organization as a member. In this case, the person with dementia analyzed his capability and options, then independently made decision.

“*Not that I don’t want, but I can’t do it in my condition now. I will still support them in many ways, but not as the leader*.” (Person with dementia, Indonesia)

The social roles changing of persons with dementia eventually impacted their direct social network and social participation. The example of impact was expressed by a family carer and a person with dementia who decided to change their social roles.

“*It used to be my husband who is active in the community, now, I need to replace him, because one of the members of the family need to join. It is our social obligation*.” (Wife of person with dementia, Indonesia)

#### 3.3.3 Ability to actively participate in social activities

Persons with dementia described the engagement in social activities such as keeping their usual habit to attend social functions on their local communities. Persons with dementia mentioned that these engagements positively impacted their mental health. Persons with dementia mentioned that the decision to participate often was driven by to their need to be productive and meaningful. For example, in one of our cases, the person with dementia was still keeping up her/his ability in writing. This made her very satisfied.

“*I also write about my experiences in my dementia situation. In good days, I can concentrate a bit for two to three hours. When I read the things three days later [.] I think ‘Wow cool, you did a great job’. I’m always really happy*.” (Person with dementia, Germany)

Some persons with dementia eventually decided to withdraw from social activities or from the interaction with loved ones due to the decline of their cognitive functions.

“*I lost a lot of friends, because it was difficult for them to talk to me and that upset me. I withdrew from social contacts*.” (Person with dementia, Poland)

“*So right now, he’s a lot quieter and more withdrawn than he was previously. I mean, he was always a reasonably quiet guy, but he was maybe just a bit more interactive and getting involved in things, whereas now he’ll kind of sit on the sidelines. I guess by natural preference and same with the kids. I think probably previously, he would have just been a little bit more with them and asking them things when now he just sits back a little bit more*.” (Daughter of person with dementia, Australia).

The reasons to join or not participate in social activities varied. Some persons with dementia explicitly mentioned how their cognitive decline negatively impacted their engagement in social activities or in conversations independently.

“*I’m also getting tired of the conversation itself [….] it’s hard for me to think and stay sharp, try to listen.*” (Person with dementia, The Netherlands)

### 3.4 Social environmental level

#### 3.4.1 Structure of social network

The direct social network confirmed that the structure of social network influenced the perceived social support of persons with dementia. Proximity, the distance to network members, seemed to be important. Some persons with dementia mentioned feelings of loneliness due to physical distance when none of their family members lived nearby. On the other hand, some persons with dementia reported enjoying living in a small village where everyone knows each other and in which they can lean on ties developed in the past from before they got dementia. They and their families appreciated being embedded in the community.

“*He (person with dementia) is still going to the mosque alone every morning. We are not worried because it is close to our house. Even if he is lost, then our neighbors will take him back home*.” (Wife of person with dementia, Indonesia)

#### 3.4.2 Function of social network

Persons with dementia acknowledged several types of support provided by the direct social network. They reported instrumental or practical support, for example, an offer to help with grocery shopping, accompanying to visit a physician or an offer to walk their dog on their ‘bad days’. Persons with dementia reported that this kind of practical supports reduced their burden knowing that they have somebody to rely on. Persons with dementia also identified emotional support. This was offered by the direct social network through various ways including sharing experiences like looking at pictures together with friends. The use of humor was also frequently mentioned by persons with dementia and the direct social network and identified as emotional support. Both parties confirmed that humor was helpful to break the boundaries and tension of the situation.

“*These days the names of the people I just forget them. And I just say: ‘I’m so sorry about that, but I forgot your name again’. I try to do a lot with humor and sarcasm and obviously people don’t hold it against me and those who did just went away.*” (Person with dementia, Germany)

Further, the direct social network mentioned how they empowered the persons with dementia to stay socially connected and contribute to daily life.

“*If there is something he can do, then we let him do it. I said: ‘Here, Dad, can you help me carry something or so.’ Not because I necessarily need it, but just so that he feels a bit needed*.” (Daughter of person with dementia, Germany)

The social network reported various resources of support. Apart from the direct network, family caregivers indicated support from neighbors and the community.

The direct social network reported what they did aiming to preserve persons with dementia’s dignity. They mentioned that they were aware that persons with dementia lost some skills and functions and gently corrected when needed. The direct social network bolstered person with dementia’s self-esteem by continuing to ask permission and advice.

“*I still discuss things and ask for his (person with dementia) permission and advice although I do not know if he can understand it properly*.” (Wife of person with dementia, Indonesia)

Sometimes the person with dementia observed such protective behaviors from the direct social network.

“*I think she [wife] shields me a bit. In terms of um … she’ll know when I’m struggling, I think, and so she would just … shield me you know, just different chatter … different talk*.” (Person with dementia, Australia)

Maintaining dignity could also result from the social network normalizing dementia as persons with dementia, so that they do not feel too bad about their condition, for instance by assuring the persons with dementia that forgetting something is a ‘normal thing’ for older persons.

“*And I just tell her, ‘It’s just it’s just age’ (person laughs), you know. Yes, yes. So anyway, that’s it*.” (Friend of person with dementia, Australia).

On the other hand, some persons with dementia mentioned that they were experiencing some non-constructive actions, which could be active (criticizing) and more implicit (avoiding, ignoring). One person with dementia felt like being ‘*constantly criticized over doing something wrong or not good enough or not listening’*. Persons with dementia also reported that the social network avoiding contact. Further, persons with dementia experienced that they were not involved in a conversation during a visit to the physician. Persons with dementia revealed that these non-constructive actions can be challenging for them. which made them feel not taken seriously.

“*I have experienced so many times that as soon as my daughter sits next to me, the doctors act as if I am not even there*.” (Person with dementia, Germany)

Health care professionals described observations of similar non-constructive actions from social network sometimes.

“*The caregiver forgets that the patient is sitting next to her, it limits her. Talking about the patient in the third person, when she is sitting next to him, takes away some of her dignity, she (person with dementia) is treated like a child*.” (Health care provider, Poland)

#### 3.4.3 Appraisal of relationships

Persons with dementia indicated the quality of relationships within their social network to be important. They mentioned some gestures of affection by family and friends, but also from their pets, to be meaningful for them.

“*Dori is a great dog. She is also a support in my life. On the day when I’m not feeling well, I look at her or when she’s lying there next to me … and that’s also such a stable factor in my life.*” (Person with dementia, Germany)

“*She obviously relies on me an awful lot and so. That’s sort of nice, in a way. Uhm, it’s it’s … She appreciates what I do for her, you know. Uhm? We, uh, we give each other lots of hugs (chuckles)*.” (Partner of person with dementia, Australia).

Persons with dementia reported that the degree of closeness with the social network influenced the quality of relationship. They mentioned that when the quality of relationship was satisfying, they enjoyed the social interaction, despite their functional challenges.

“*We have very nice neighbors. We always go out for dinner and so on. Of course, I can’t say anything because I’m not that fast [fast here could mean = I am a bit slow]. But that’s always nice*.” (Person with dementia, Germany)

Both persons with dementia and the direct social network emphasized that the affection that is shown to each other is highly appreciated by both parties.

“*What is very important is the way my daughters deal with me, their love, and affection*.” (Person with dementia, Germany)

## 4 Discussion

The current qualitative study contributes to the identification of social health markers from the perspective of persons living with dementia and their direct social network, including their primary informal and professional caregivers and other social network members. The current study also identified some social health markers that have not been used in epidemiological nor intervention studies. It led to the refinement of and therewith better understanding of the social health domains as defined in the recently developed framework (18). Interviews also revealed how persons with dementia used their remaining talents and skills to comply with social norms and to live their lives as usual.

In terms of individual level social health markers, we found that the person with dementia’s capacity was impacted by cognitive and functional changes to which they tried to adapt continuously. Autonomy was expressed by making choices in keeping contact or withdrawing socially, and accepting or declining support being offered. In general, being involved in decision making in daily life played a key role in preserving autonomy. We found a variety in degrees of participation in social activities as well as in reasons to join in activities. Persons with dementia mentioned that engaging in activities provide positive impact on their mental health.

In addition to traditional markers of social network structure such as marital status, living alone or lack of social contact, we found that proximity (physical distance) indeed is a relevant structural marker. Physical distance caused persons with dementia to feel isolated. Some persons with dementia who lived in a small village, where people know each other, felt more closely related to their social network in which they could lean on contacts from the time before they got ill. Previous research mentioned that living with dementia in rural area enhances the sense of self-sufficiency of persons with dementia (31). The neighborhood is perceived as a place with biographical attachment and emotion connections with familiar friends and relatives (32, 33) as well as a relational place for persons with dementia to engage and interact with familiar faces (34). In fact, the value of proximity for persons with dementia was the fundamental idea behind some programs such as dementia-friendly communities (35). This confirms the (more implicit) acknowledgement of this specific marker. Future studies may inform us on how to intervene on this specific marker to make persons with dementia in more urban areas obtain social health value from their neighborhood.

With regards to social network functions, we found that emotional support by using humor and sharing experiences were appreciated by both persons with dementia and the direct social network. The direct social network often supported persons with dementia to maintain their dignity, by active and more implicit empowerment. However, we also identified some non-constructive actions like not involving persons with dementia in conversations. Relevant markers on the appraisal of the relationship were affection and appreciation for example closeness of relationship and sharing common ground.

We found that persons with dementia attempted to live as normal as possible and had intentions to comply with social norms. These findings are consistent with previous literature. Review studies pointed out that persons with dementia aim to maintaining a normal situation in daily life (36) and continuity in their lives as a coping strategy (37). As a result of the fear of not being able to comply with social norms in social interactions, persons with dementia tended to exclude themselves from social participation (38). Therefore, health care professionals underlined the importance in maintaining ordinariness in life of persons with dementia to support their intention to comply with social norms (39). Whereas previous research showed that the level of persons with dementia’s empathy during their trajectory of illness is declining (40), we found that many of them still desired to show empathy to others. Efforts of persons with dementia to live life as normally as possible has been described in previous literature (36, 37) but not explicitly been labelled as an attempt to comply with social norms, obligations or tasks. We hypothesize that persons with dementia’s intention to live life as usual can be due to their intention to comply with social norms and the fear of being excluded. The complex interplay within these two relevant markers needs to be further explored in the future studies. For the most relevant markers identified, we discuss their relevance and novelty in relation to the existing literature below.

Our data showed that persons with dementia used their ability to manage life with some degree of independence for keeping contact and activities as well as to accept or refuse support being offered. Therewith, persons with dementia focused on autonomy with regard to daily life decisions rather than major end-of-life decisions such as advance care planning. This corresponds with the findings from several qualitative studies which found that persons with dementia are still maintaining their autonomy during the disease trajectory (41), especially to participate in making choices in everyday life (42). Even in the context of advance care planning conversations, persons with dementia highly appreciated and preferred discussing short-term non-medical topics (43) over deciding on advance directives. This emphasizes how the little things in life influence the quality of life of older adults (44, 45).

We found that the direct social network can influence persons with dementia in both positive and negative ways. Our study revealed that the most powerful positive approach was for family caregivers and other social network members to actively promote dignity of persons with dementia. This specific marker was found in all participating countries which illustrates its relevance, regardless of cultural background. Previous review studies pointed out that the social network helps preserving persons with dementia’s dignity by offering personalized care such as implementing patient centered care or by showing respect with involving persons with dementia during care process (46, 47). When persons with dementia are no longer able to make sound decisions for themselves, dignity still can be preserved by employing some persuasive actions (47).

We also found negative functioning of social network and non-constructive attitudes toward persons with dementia such as excluding them during conversations. This very active exclusion contradicts with previous studies that merely provide illustrations of passive-non-constructive attitude such as people being afraid to be around and avoiding persons with dementia although they have sufficient knowledge on this illness (48–50). Overall, these negative approaches emphasized how social health of persons with dementia is threatened by stigma. Stigma is a complex concept and varies amongst ethnic and culture (51) and widely described in relation to dementia (52). Stigmatization by the social network was shown to lead to self-stigma at the individual level, manifested in our study and confirmed in the literature by withdrawal from activities due to feelings of incompetence (52).

Our study provided empirical data on social health markers as well as identified new markers that have not used in previous epidemiological nor intervention studies. Next, future research is needed to unravel the interplay between markers within and between domains and levels and to study the association between social health as a concept, and cognitive decline and dementia. Further, to be able to study novel social health markers such as autonomy-in-everyday life, the development of instruments is essential.

### 4.1 Strengths and limitations

The current study identified social health markers which were underexplored in extant dementia research that examined the association between social health and cognition/dementia. Secondly, we started with open coding and used the conceptual framework of social health in dementia to organize the codes (18). The fact that our inductive codes fit within this framework, underlines the credibility of our findings and of the framework. Thirdly, in order to provide uniformity of the data collection process, we used the same data collection method and topic guide in all participating countries. Also, we applied investigator triangulation for data analysis and interpretation, as we included multidisciplinary perspectives from our international research team which come from various educational backgrounds, namely sociologists, psychologists, neuropsychiatrists, geriatricians and nursing.

The current study has a number of inherent limitations/challenges due to the involvement of multiple countries and languages. The transcripts were drafted in original languages, but the codes were generated in English, which may have led to details getting ‘lost in translation’. In order to limit this loss of credibility, we managed to have at least two independent coders in each participating country. We iteratively developed a codebook including definitions with all partners from all different countries to facilitate the harmonization of the codes. Further, as we were restricted by ethical regulations in data sharing from each country, we shared and discussed data on the coding level. As a consequence, no single researcher had access to all the transcripts. This may have led to heterogeneity in the interpretation of the codes. As mentioned above, the researchers did, however, discuss the harmonisation and scope of each of the codes to minimise coding heterogeneity.

The current study also faced the reluctance of involvement of primary health care professionals. Health care professionals can be seen as ‘observer’. Therefore, the consequence of lacking healthcare voices is that in some cases we may have lost the “objective” voice in the description of examples of interaction between person with dementia, family and active network member.

## 5 Conclusions

The current international qualitative study identified and articulated novel social health markers both on the level of the person with dementia and on their social environment, that provide further insights into the recently established social health conceptual framework. Our results added refinement to the domains within the framework by the identification of new sub-domains as ‘dementia symptoms limit social capacity’ and ‘adaptation’ within the individual level. Instrument development and validation are essential next steps to enable measurement and implementation in ongoing cohort studies. This may provide further understanding of these markers and their interplay. Understanding the interplay between social markers may contribute to a more complete multifaceted perspective on the entire dementia patient journey from first symptoms to advanced disease. When used as a fundament in innovative intervention studies, these markers may ultimately contribute to developing interventions focused on preventing dementia and on living a meaningful life for persons with dementia and their social network.

## Data Availability

The original contributions presented in the study are included in the article/**Supplementary Material**. Further inquiries can be directed to the corresponding author.
