# Supplementary material for: Social health markers in the context of cognitive decline and dementia: an international qualitative study
Source: Front Psychiatry. 2024 Sep 19;15:1384636. doi: 10.3389/fpsyt.2024.1384636 (PMC11448353; doi:10.3389/fpsyt.2024.1384636)
Supplement: Supplementary file 1 [file DataSheet1.docx]

Appendix 1. Interview guide

1. **Dementia diagnosis**

- Can you explain what dementia means to you?
- What is the impact of your disease on your functioning? (physical, social, psychological and cognitive function)
- How do you deal with the consequences you experience as a result of dementia?
- What kind support do you receive and from who?
- How do you experience the support that you receive?
- What is pleasant with regard to receiving support?
- What is less pleasant about receiving support?

1. **Social network and daily activities**

- How many people do you see during the day (did this change before and after the diagnosis)?
- To what extent are you engaged in joined activities such as shopping, walking, membership of clubs?
- What kind of activities do you enjoy? Are you satisfied with your daily activities and functioning? Are there persons with whom you prefer to discuss

1. **Influence of social network on functioning**

- When do you feel you are able to fulfill your obligations/tasks such as E.g. selfcare, taking care for others, using your skills and capacities?
- When do you feel being stimulated? When do you feel being discouraged?
- When do you have the feeling being valued by others? How do you experience that your contributions are appreciated?
- What reactions are helpful and unhelpful in managing the condition?

1. **Changes in social network and interaction with social network after dementia diagnosis**

- When do you withdraw from activities or interactions?
- To what extent do you have the feeling being treated differently after the diagnosis? When do you feel neglected and less valued? When do you feel being appreciated?
- What is the influence of the interaction with the person with dementia on your [informal caregiver/healthcare professional/key social network
